# Treatment of Prepubertal Labial Adhesions with Topical Estriol + Testosterone: A Case Report

**DOI:** 10.3390/pediatric16030047

**Published:** 2024-07-12

**Authors:** Filippo Murina, Cecilia Fochesato, Valeria Maria Savasi

**Affiliations:** 1 Lower Genital Tract Disease Unit, V. Buzzi Hospital, University of the Study of Milan, 20124 Milan, Italy; cecilia.fochesato@unimi.it; 2 Department of Woman Mother and Neonate, V. Buzzi Hospital, University of the Study of Milan, 20124 Milan, Italy; valeria.savasi@unimi.it

**Keywords:** labial adhesions, labial agglutination, prepubertal, estrogen, betamethasone, testosterone

## Abstract

Background: Labial adhesions, a frequent gynecological condition in prepubertal girls, occur when the labia minora adhere along the midline. The prevailing hypothesis about their etiology suggests that labial adhesion may occur when the delicate and non-estrogenized labia minora undergo an inflammatory response, triggered by exposure to an irritant environment. Therefore, conservative treatment involves the application of topical estrogen or betamethasone cream. The role of androgens has not been considered yet in the pathophysiology or therapy of this condition. However, some studies have shown that androgen receptors are prevalent in the labia minora and vulvar vestibule. Case summary: We present the case of a 29-month-old girl with symptomatic labial adhesions. She was first ineffectively treated with topical estriol, and then she was treated with a galenic cream containing both estriol and testosterone with complete recovery and without side-effects. Conclusions: Both androgens and estrogens play a significant role in maintaining the physiological trophic state of the vulva and vagina, even during childhood. Topical estriol+testosterone could be considered an alternative treatment for prepubertal labial adhesions refractory to standard topical therapy.

## 1. Introduction

Prepubertal labial adhesions, alternatively named labial agglutinations or labial fusions, represent an acquired medical condition, characterized by the adherence of the labia minora in the midline [1]. Usually, the fusion starts from the caudal to ventral side of the labia minora and could progress until only a little opening is left anteriorly (through which urine can pass with struggle) [2]. The diagnosis is made through a close clinical inspection of the genital area usually in the “frog leg” position [3]. It is noteworthy that the real prevalence is challenging to assess, given the current lack of universally accepted diagnostic criteria and the common absence of symptoms. The widely acknowledged estimated occurrence of labial adhesions is 0.6–3% among prepubertal girls [1], which are typically absent in newborn infants [4], and their incidence reaches a peak of 3.3% between 13 and 23 months of age [4]. However, some authors describe a higher prevalence, reaching up to 38.9% [5,6].

The etiology of this condition is not entirely clear, and the general theory is that labial fusion can occur when the vulnerable and thin non-estrogenized labia minora develop an inflammatory reaction due to the exposure to a local irritant setting [1,7,8].

Labial fusions are often asymptomatic. Otherwise, if symptoms are present, the most reported are urinary tract infections, abnormal bladder voiding, vaginitis, vaginal discharges, or vulvar pain [1,8,9].

Currently, no specific therapy is recommended for asymptomatic patients, because a lot of them have a spontaneous resolution, especially during puberty [3,8]. On the other hand, treatment is mandatory if girls are symptomatic [1,8,10] or, for some authors, even if parents are extremely concerned about the appearance of the genitalia of their children [9]. Traditionally, considering hypoestrogenism and chronic inflammation as causatives, the first-line therapy for labial adhesion has been topical estrogen cream [8,10,11,12,13,14,15,16,17,18,19,20,21,22,23,24,25] or an alternative topical betamethasone cream [9,10,13,17,26]. More invasive treatments like manual or surgical separation are applied only in selected cases [2,27].

In general, topical hormonal therapy is used in the vulvovaginal area for the treatment of a lot of different conditions, and specifically considering the anatomy of the labia minora, some studies demonstrated that in this area, estrogen receptors are present, but androgen receptors are prevalent [28,29].

Consequently, topical vaginal and vulvar treatments with estrogen in combination with testosterone are already used as therapy for the genitourinary syndrome of menopause [30,31,32]. Recent observations, particularly in animal studies, suggest that androgens may exert a direct influence on the structure and function of the vulvovaginal area, independent of estradiol [31]. The effectiveness of testosterone in treating vaginal atrophy has been attributed to collagen neoformation, vasodilation, and increasing mucus secretion [31]; in our opinion, these effects can be useful even in the treatment of prepubertal labial adhesions. Otherwise, as we know, a combination of estriol and testosterone was never used to treat labial adhesion in prepubertal girls.

We present the case of a girl who underwent clinical examination at our vulvar pathology outpatient clinic for symptomatic labial adhesions. After first-line treatment with topical estriol cream with limited results, she was treated with a galenic cream containing estriol and testosterone with a complete recovery and without any side-effects.

## 2. Case Presentation

An Italian 29-month-old girl was referred to our vulvar pathology outpatient clinic by her pediatrician for symptomatic vulvar adhesions.

At the time of the visit, the child’s development appeared appropriate for her age.

In her past medical history, she did not mention any significant illness, surgical procedure, or history of sexual abuse. The girl was still using diapers and her parents reported adequate daily intimate hygiene, using specific products. Her mother noticed a partial fusion of her daughter’s labia minora that started six months ago and progressively became more evident. The girl did not complain of any burning sensation, itching, or atypical vaginal discharge. However, she had two episodes of urinary tract infection characterized by frequent urination and low-grade fever, treated with specific antibiotic therapy, and resulting in complete recovery.

During our medical assessment, the general physical examination was normal. Therefore, we informed parents and patients of the need for an inspection of the external genitalia, with possible photographic documentation. The child was positioned lying on the examination bed, with the mother beside her, and examined in the “frog-leg” position.

**Figure 1:**
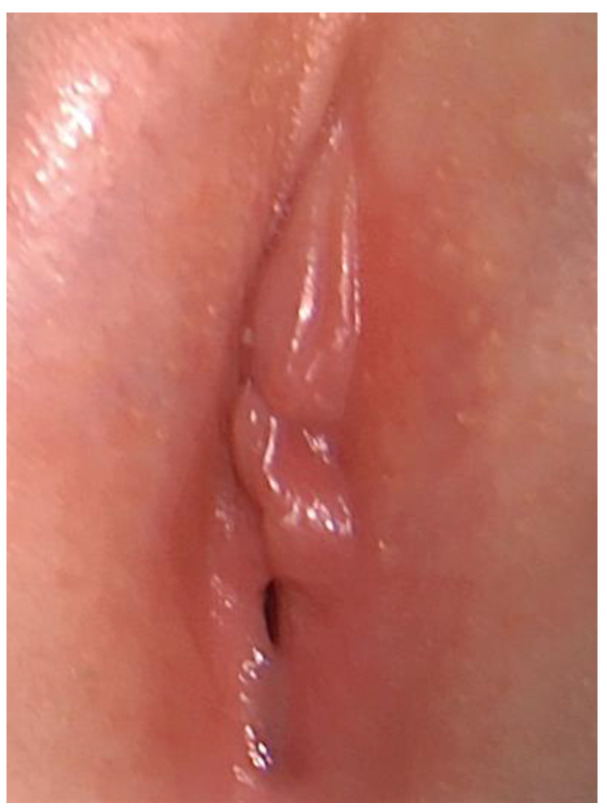
Labial adhesion at the diagnosis.

Upon inspection, the labia majora were glabrous and normal for age. Moreover, the lower 2/3 of the labia minora appeared fused, causing a sub-stenosis of the vaginal introitus, and the urethral meatus was only partially visible. With gentle lateral traction of the skin, a translucent area of stickiness was evident at the point of labial fusion, leading to a diagnosis of prepubertal labial adhesion (Figure 1).

Firstly, we decided to administer estriol ointment 50 mcg/g. The parents were instructed to apply every night before bedtime almost half of a fingertip unit (almost 0.25 mL) of cream directly on the adhesion using a cotton swab, after adequate intimate hygiene. 

**Figure 2:**
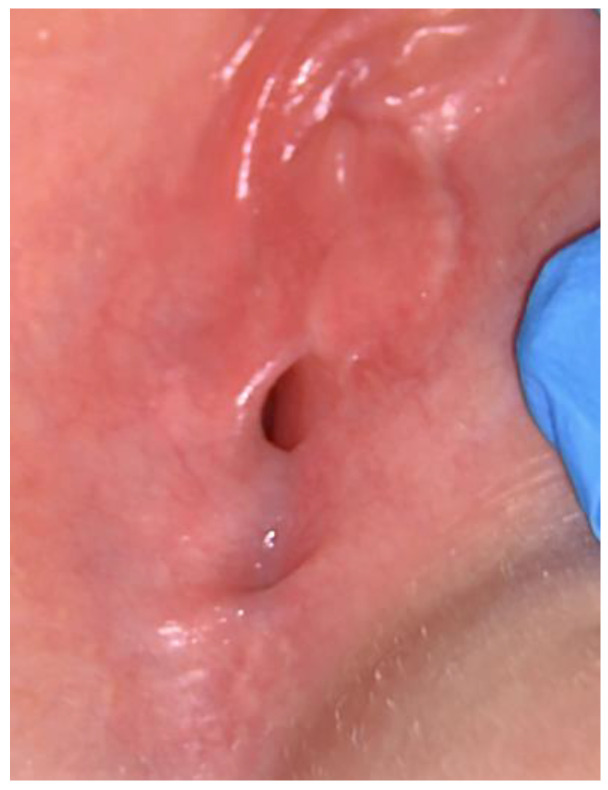
Labial adhesion after estriol cream therapy.

The patient returned for a check-up after 21 days in good general condition, without reporting any side-effects of the treatment prescribed. Inspecting the external genitalia, only a minimal reduction in the stenosis was noted, with a vaginal opening between the labia minora of only 4 mm (Figure 2).

Considering the inadequate response to the previous therapy, we decided to administer, through a Topi-CLICK^®^, a galenic topical cream already used in patients with genitourinary syndrome of menopause. This ointment contains testosterone vegetal oil 0.28%, estriol 0.05%, Argan oil 1 g, and Pentravan 0.98 g/mL. Topi-CLICK^®^ is a precision-dosing dispenser that is easy to use and helps to achieve maximum compliance with medication. The parents were instructed on how to apply the ointment amount of 0.25 mL (corresponding to one click of the Topi-CLICK^®^), with a cotton swab, following the same previously described modalities, for 28 days.

**Figure 3:**
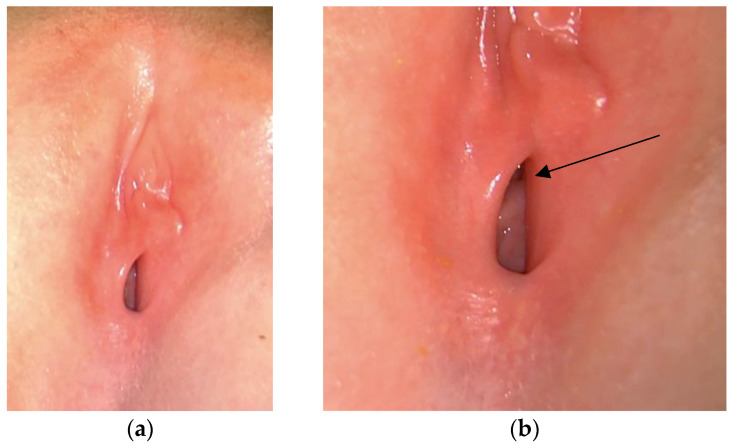
Labia minora after testosterone+estradiol cream therapy. Normal vaginal opening is visible (**a**). The arrow indicates the urethral meatus (**b**).

At the subsequent follow-up, the patient showed a complete recovery of the adhesion with a regular aspect of the labia minora and a normal vaginal opening (Figure 3a,b). There were no signs of local irritation and systemic hyperandrogenism or hyperestrogenism. 

At the 3-month follow-up, there were no reports of the recurrence of labial adhesions, and no additional signs or symptoms were observed.

The compliance of the parents and the patient was optimal, and when the family was questioned about any issues encountered during the administration of the therapy, they reported no difficulties or unwanted effects.

## 3. Discussion

Labial adhesion is a condition characterized by the fusion of the labia minora over the vestibule, usually progressing from the caudal to the ventral part of the labia minora. The diagnosis is made with the identification of a thin avascular membrane in the midline. Labial fusion is rarely observed at birth, and it is predominantly considered an acquired condition [4]. This condition represents a relevant disorder in the female pediatric population with an estimated occurrence of 0.6–3.3% in prepubertal girls [4,7]. However, some authors describe a higher prevalence, reaching up to 38.9% [5,6]. In our opinion, this variety in incidence is firstly related to the lack of universally accepted diagnostic criteria, and secondly, it is conditional on the limited data collection of asymptomatic patients.

Indeed, symptoms vary, depending on the extent and severity of the agglutination. Most girls are asymptomatic and are referred to the gynecologist due to parental or primary care provider concerns regarding an absent or closed vagina. Instead, in symptomatic patients, common complaints include vulvovaginal irritation, pain during wiping and urination, discomfort with certain movements (e.g., straddle activities), or postvoid dribbling. In more severe cases, symptoms may involve recurrent urinary tract infections or urinary retention [7].

The physiopathological base of labial adhesions is not completely clear, and many authors postulated that they could develop in girls exposed to local irritants in a low-endogenous-estrogen setting, characterized by the thin and immature epithelium of the labia minora [7]. This scenario is more likely to occur in infants and young children who wear diapers, because the labia minora are frequently in close opposition, especially in the posterior region. It is widely recognized that children typically transition beyond diaper usage after the age of 2–3 years old, leading to a reduction in diaper dermatitis and vulvovaginitis that can create chronic inflammation. After 2 years old, children also become more active, frequently engaging in sitting and standing activities throughout the day, resulting in a more frequent spontaneous opening of the labia minora. These considerations might explain the peak incidence of labial agglutinations of 3.3% between 13 and 23 months of age [4]. 

The treatment of labial adhesions is usually reserved for symptomatic girls as a lot of asymptomatic cases will resolve spontaneously, especially with the onset of puberty [1,3,7,8]. A recent retrospective study supported the resolution of labial adhesions without treatment in over 40% of occurrences after a median follow-up of 3.6 years [3]. Several authors recommend, even in asymptomatic cases, the implementation of hygiene measures, which involve the elimination of all potential vulvovaginal irritants, including soaps, bubble baths, and restrictive clothing, along with the incorporation of daily sitz baths [1].

The first-line treatment for symptomatic labial adhesions in prepubertal girls is considered the use of topical estrogen ointments, applied with gentle pressure on the adhesion line using a fingertip or a cotton swab. Therefore, topical estrogen therapy has demonstrated greater efficacy compared to the wait-and-see approach [25].

In the literature, a high variability of estrogen cream efficacy is reported, from 15.5% to 100%. These discrepancies could mainly be attributed to the lack of a universal definition of labial adhesion with clear diagnostic criteria. Even different schemes of administration (once or twice daily), different extensions of treatment (from two to nine weeks or even more), and different kinds of topical estrogens used (conjugated estrogens 0.625 mg/g “Premarin”, dienestrol 0.01%, estradiol 0.01% “Estrace”) influence the overall efficacy [8,10,11,12,13,14,15,16,17,18,19,20,21,22,23,24,25]. Side-effects of topical estrogen therapy are rare and may include local irritation, vulvar hyperpigmentation, and breast budding, but the discontinuation of therapy always resulted in their complete resolution [7,25].

An alternative conservative treatment for labial agglutination is the use of topical steroids [9,26]. Corticosteroid creams (betamethasone dipropionate 0.05% or betamethasone valerate 0.1%) are applied one or two times a day for 2–12 weeks, with an average success rate reported in the literature between 15.6% and 89.5% [9,10,13,17,26]. In our opinion, even in this case, the variability in effectiveness is influenced by the different drug administration schemes and by the lack of uniformity in labial adhesion diagnostic criteria. Side-effects of topical steroids are uncommon and may include erythema, folliculitis, and low-grade skin atrophy [7].

To unify the definition of labial adhesion and reduce the heterogeneity of the efficacy results reported in the literature, in a recent study, Huseynov et al. tried to classify labial agglutinations on the severity of presentations. They found a strong correlation between the different subtypes and the efficacy of treatment [9].

Refractory symptomatic adhesions or those causing complete urinary obstruction may necessitate manual or surgical mechanical separation. The procedure can be performed with topical lidocaine or under general anesthesia and should be referred to an experienced provider. The manual separation of asymptomatic and uncomplicated adhesions is discouraged, as procedure-related inflammation may increase the risk of recurrent and more resistant adhesions [7,8].

Recognizing the key role played by topical medical therapies in the treatment of labial adhesions, it is important to try to understand their physiological rationale. As we reported before, the etiology of labial agglutination is usually attributed to a chronic inflammatory process acting on the thin non-estrogenized epithelium of the labia minora.

The low serum estradiol levels detected in infants between 3 months and 5 years old can potentially explain the higher frequency of labial fusion during these age ranges [33]. However, in 2003, Papagianni et al. reported the co-occurrence of isolated premature thelarche and labial adhesions [34]. It is widely known that a premature thelarche is associated with elevated estrogen levels in young girls, which contrasts with the hypoestrogenic theory for the onset of labial agglutinations. Furthermore, a comparative study achieved in 2007 disproved the correlation between low serum estradiol levels and labial adhesion [33]. These findings can suggest the involvement of other factors besides hypoestrogenism in the etiology of labial adhesions and even other reasons for their apparent efficacy in therapy [33]. According to some studies, topical estrogens can accelerate the cutaneous wound healing process and re-epithelialization-enhancing matrix deposition, reducing the inflammatory response and stimulating the production of Heparin-binding epidermal-like growth factor (HB-EGF) in keratinocytes [24,33,35]. These findings can help to explain the efficacy of estrogen topical therapy on labial agglutination.

According to other authors, chronic inflammation is the major determinant in the etiology of labial adhesion; therefore, they propose topical steroids as a treatment option. In 2006, Myers et al. were the first to propose this shift in topical therapy, comparing the etiology of male phimosis to labial adhesions [26]. According to them, inflammation, especially associated with diaper use, creates microtrauma on the labia minora skin that, during the re-epithelization process, can lead to the development of labial fusion. For this reason, some authors consider steroid therapy as the most appropriate treatment for labial agglutination, with fewer associated side-effects [10,13,26].

In our opinion, neither of these two theories fully explains the etiology of labial adhesions, and the role of androgens should also be taken into consideration.

Immunohistochemical and histological research has demonstrated that the amount of androgen receptors in the labia minora, labia majora, and vestibule is higher than that of estrogen receptors [28,29]. Considering female genitalia development, the embryological origin of the labia majora, labia minora, and vagina is different. The urogenital sinus forms the vestibule of the vagina, the two urogenital folds of the genital tubercle develop into the labia minora, and the labioscrotal folds enlarge to create the labia majora [28]. These distinct embryological origins can partially account for the different concentrations of estrogen and androgen receptors in these structures. In addition, plasmatic estradiol and testosterone concentrations follow the same pattern; during early childhood, they are low and generally gradually increase during prepuberty, and then they decrease in menopause [28,36]. Therefore, a deficiency in androgens may also be temporally correlated with the onset of labial adhesions.

The effect of androgen receptor activation is associated with cell proliferation, differentiation, metabolism, and apoptosis, as well as protein secretion in various tissues in both men and women [28]. Some studies on genitourinary syndrome in menopause suggest that testosterone may have a direct and estrogen-independent effect on the vaginal and vulvar structure as evidenced by improvements in mucous secretions, vasodilation, and the deposition of new collagen with testosterone topical therapy [30,31,37].

In this case report, we presented a girl suffering from labial adhesion, who was treated, at first, ineffectively with topical estriol, and secondly, with a galenic cream containing testosterone vegetal oil 0.28%, estriol 0.05%, Argan oil 1 g, and Pentravan 0.98 g/mL with complete resolution. In our opinion, the simultaneous use of testosterone and estriol, with adjuvant Argan oil and Pentravan, is key to the effectiveness of our therapy. The application of topical estrogens, as previously described, has the potential to promote re-epithelialization by augmenting matrix deposition while concurrently diminishing the inflammatory response. However, the amount of estrogen receptors in the labia minora is lower than that for androgens. For this reason, the association with testosterone can improve mucous secretions, vasodilation, and the deposition of new collagen fundamentals for a complete recovery. An adjuvant effect is also exhibited by Argan oil and Pentravan. Some studies demonstrated that the daily topical application of Argan oil improves skin elasticity and hydration by restoring barrier function and maintaining water-holding capacity [38,39]. Moreover, Pentravan, an oil-in-water vanishing cream base, can facilitate the accumulation and transdermal delivery of topical hormonal drugs [40].

In our opinion, an ointment containing both estrogens and androgens should be considered for the treatment of labial adhesions. Further studies are needed to assess if the addition of topical testosterone can enhance the efficacy and reduce the recurrence of labial adhesions without significant side-effects.

## 4. Conclusions

Prepubertal labial adhesions are an underestimated pathology with a complex physiopathology, probably multimodal. Both androgens and estrogens play a major role in the maintenance of the vulvar and vaginal physiological state, even in childhood.

Although little is known about the function of androgens in the genital apparatus of women, specifically in infancy, the abundance of androgen receptors in the labia minora, labia majora, and vestibule must be considered. The enhancement in the knowledge of the role of androgen can help us improve new therapeutic strategies for different vulvar conditions, including prepubertal labial adhesions.

## Data Availability

Data are contained within this article.
